# Phlorotannins: Towards New Pharmacological Interventions for Diabetes Mellitus Type 2

**DOI:** 10.3390/molecules22010056

**Published:** 2016-12-30

**Authors:** Graciliana Lopes, Paula B. Andrade, Patrícia Valentão

**Affiliations:** Requimte/LAQV, Laboratório de Farmacognosia, Departamento de Química, Faculdade de Farmácia, Universidade do Porto, Rua Jorge Viterbo Ferreira, No. 228, Porto 4050-313, Portugal; gracilianalps@gmail.com (G.L.); pandrade@ff.up.pt (P.B.A.)

**Keywords:** phlorotannins, diabetes mellitus, hyperglycaemia, α-glucosidase, postprandial glucose, advanced glycation end-products, pancreatic β-cells, glucotoxicity

## Abstract

Diabetes mellitus is a group of metabolic disorders characterized by hyperglycaemia, and predicted by the World Health Organization as the expected 7th leading cause of death in 2030. Diabetes mellitus type 2 (DMT2) comprises the majority of diabetic individuals around the world (90%–95%). Pathophysiologically, this disorder results from a deregulation of glucose homeostasis, worsened by overweight and by a sedentary lifestyle, culminating in life-threatening cardiovascular events. The currently available anti-diabetic drugs are not devoid of undesirable side effects, sometimes responsible for poor therapeutic compliance. This represents a challenge for contemporary medicine, and stimulates research focused on the development of safer and more efficient anti-diabetic therapies. Amongst the most promising sources of new bioactive molecules, seaweeds represent valuable, but still underexploited, biofactories for drug discovery and product development. In this review, the role of phlorotannins, a class of polyphenols exclusively produced by brown seaweeds, in the management of DMT2 will be discussed, focusing on various pharmacologically relevant mechanisms and targets, including pancreatic, hepatic and intestinal enzymes, glucose transport and metabolism, glucose-induced toxicity and β-cell cytoprotection, and considering numerous in vitro and in vivo surveys.

## 1. Introduction

Diabetes mellitus (DM) is a heterogeneous disease with a considerably high variation in clinical presentation and progression. According to the American Diabetes Association, DM can be defined as a group of metabolic diseases characterized by hyperglycaemia resulting from defects in insulin secretion, insulin action, or both [1]. Recent data from the World Health Organization (WHO) points to 422 million people suffering from diabetes in 2014, which represents a rise of 314 million cases since 1980. This dramatic increase in prevalence makes the WHO predict diabetes as the 7th leading cause of death in 2030 [2].

Diabetes classification is not always easy and depends on the circumstances at the time of diagnosis, being more accurate over time. Three general categories can be distinguished: diabetes mellitus type 1 (DMT1), diabetes mellitus type 2 (DMT2) and gestational diabetes mellitus (GDM). Other specific types of diabetes, such as impaired glucose tolerance (IGT) and impaired fasting glycaemia (IFG), can also occur, and represent intermediate conditions in the transition between normality and DMT2 [1]. Despite the different categories, the vast majority of cases of DM fall into DMT1 and DMT2. DMT1, or insulin-dependent diabetes, affects 5%–10% of diabetic individuals, and primarily results from a cellular-mediated autoimmune destruction of the pancreatic β-cells. It affects mainly adolescents, ketoacidosis being the first manifestation. DMT2 comprises the majority of diabetic individuals around the world (90%–95%). It results from resistance to insulin and to a relative insulin deficiency, being in large part a result of overweight and of a sedentary lifestyle [2,3]. 

Overtime, the consequences of diabetes can be life threatening, damaging blood vessels, heart, eyes, kidneys and nerves. Long term complications include retinopathy with potential loss of vision, nephropathy and renal failure, peripheral neuropathy with risk of ulcers and amputation, atherosclerosis and cerebrovascular disease [3,4]. Diabetes can be treated and its consequences avoided or delayed with diet changes, physical activity, medication, and regular screening and treatment for complications. Prevention can be achieved by maintaining a healthy diet and body weight, and by effective control of blood glucose levels, which constitutes a key point to prevent or delay the disease complications and to improve patients’ life quality [5].

The pharmacological interventions to control hyperglycaemia include oral therapy with anti-diabetic agents and intramuscular administration of insulin. Different classes of oral anti-diabetic drugs are available, namely insulin secretagogues (sulfonylureas and rapid-acting insulin analogues), α-glucosidase inhibitors and insulin sensitizers (biguanides and thiazolidinediones). The monotherapy selection is based on clinical and biochemical assessment of the patient, the drug doses being titrated according to glycaemia response; dual and triple combination therapy is necessary when monotherapy is insufficient [6]. Despite their clinical effectiveness, currently available anti-diabetic drugs are not devoid of undesirable side effects, such as risk of hypoglycaemia, flatulence, weight gain and enhancement of gastrointestinal problems, which can sometimes be responsible for therapeutic non-compliance. For instance, it is estimated that the adherence to oral hypoglycaemic drugs of individuals with DMT2 is between 36% and 93%, which may represent one of the major factors contributing to sub-optimal control [7].

Marine organisms are rich sources of bioactive compounds, with diverse molecular structures and biological activities [8,9]. Their relative safety is one of the main reasons behind their exploitation in pharmaceutical, cosmetic and nutraceutical bioprospecting, placing them as privileged targets of chemical and molecular studies [10,11]. The huge diversity of marine species, and their exposure to extreme environments, stimulates them to produce a wide range of bioactive compounds through diversified metabolic pathways, placing them at the forefront as the most appealing sources of drug leads in the XXIst century [8,12]. A wide diversity of marine organisms has been explored in the search for new bioactive molecules with anti-diabetic potential [13]. Seaweeds constitute one of the most important living resources in this field, their dietary consumption being associated with lower risk of cardiovascular disease, hyperglycaemia and breast cancer. As sources of novel and structurally diverse bioactive compounds, not commonly present in terrestrial plants, the biomedical potential of seaweeds is still largely underexplored by the scientific community [14]. Despite the enormous variety of marine drugs with anti-diabetic potential explored in the last decades, in this review we will exclusively focus on and cover the anti-diabetic potential of phlorotannins. This class of brown seaweed polyphenols will be explored for their capacity to manage DMT2 via various pharmacologically relevant targets, and compared with the reference drugs.

## 2. Diabetes Mellitus Type 2

DMT2 is the major common form of diabetes, and is characterized by a combination of factors including resistance to insulin action and inadequate compensatory insulin secretory response [3]. This condition leads to a prolonged and gradual development of hyperglycaemia, not often severe enough to provoke noticeable symptoms, but sufficient to cause pathologic and functional changes in various target tissues [1]. As hyperglycaemia may be present for a long period of time before diabetes is detected, patients can remain undiagnosed for many years, being at increased risk of developing vascular complications [15]. Most of individuals with DMT2 are obese or have a high percentage of body fat distributed around the abdominal region. This condition increases insulin resistance and, consequently, originates a defective glucose uptake and hyperglycaemia. In addition to dietary carbohydrates, high glucose levels can also derive from an abnormal increase in hepatic glucose production, stimulated by abnormally high levels of glucagon secretion from pancreas. In both situations there is a stimulation of pancreatic β-cells to increase insulin production. However, since β-cell insulin secretion does not result in an immediate decrease in glucose levels, as happens in non-pathologic conditions, more insulin will be produced by β-cells as a compensatory response, leading to subsequent β-cell failure and death [1,16]. Besides hyperglycaemia, inadequate or abnormal patterns of insulin release can also be caused by pancreatic β-cell impairment.

Different strategies have been adopted over the years to help maintaining glucose homeostasis, thus avoiding disturbances in insulin metabolism and hyperglycaemia-related complications in type 2 diabetic individuals. Even though weight loss and the adoption of a healthy lifestyle can improve glucose homeostasis by increasing the sensitivity to insulin, pharmacological therapy is most often necessary. The most frequent anti-diabetic drugs are focused in preventing and reducing hyperglycaemia, and include drugs able to inhibit carbohydrate absorption, endogenous glucose production, insulin sensitizers, insulin secretagogues, insulin receptor agonists, and inhibitors of glucagon release [6,17,18,19]. Besides hyperglycaemia, some efforts are being made to control the major diabetes-related vascular complications that result from glucose-induced toxicity. In fact, the major concern in diabetes is not hyperglycaemia per se, but what results from hyperglycaemia, namely the glucose-induced toxicity and cell destruction, and protein glycation and dysfunction, that result in the associated life-threatening vascular events [20,21].

## 3. Phlorotannins

**Figure 1:**
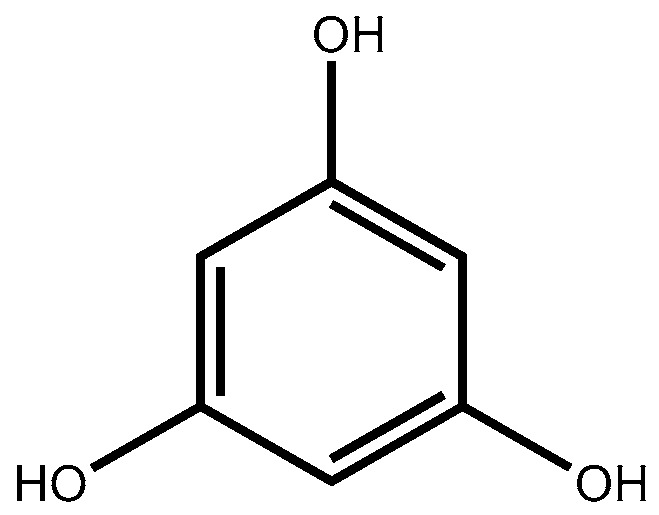
Structure of phloroglucinol.

Phlorotannins are polyphenolic compounds with a wide range of molecular weights (126 to 650 kDa), which have been reported for almost all brown seaweed orders. They are formed by the polymerization of their aromatic precursor phloroglucinol (1,3,5-trihydroxybenzene, Figure 1), through the acetate-malonate pathway, also known as polyketide pathway. The biosynthesis occurs in a process involving polyketide synthases-type enzyme complexes; the polymerisation of phloroglucinol units continues with seaweeds life time, giving origin to larger polyphenols as the organisms mature [22].

**Figure 2:**
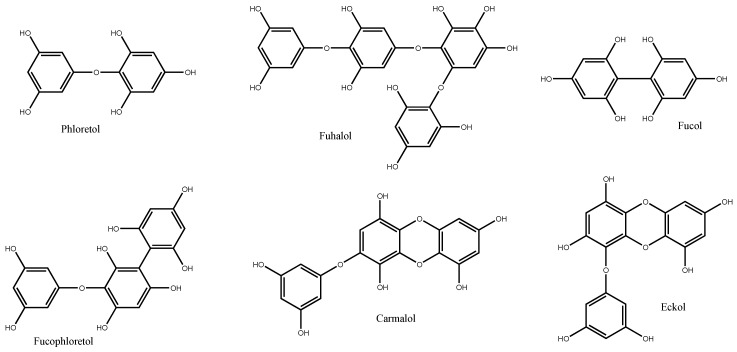
Different classes of phlorotannins [23].

Six different classes of phlorotannins can be distinguished—phlorethols, fuhalols, fucols, fucophlorethols, eckols and carmalols—based upon variations in their assemblage from the polymerization of phloroglucinol units, and distribution of hydroxyl (OH) groups (Figure 2). In phlorethols and fuhalols, then phloroglucinol units are linked by aryl ether bonds, fuhalols differing by their regular sequence of *para*- and *ortho*-ether bonds, by the presence of additional OH groups in every third ring and by the lack of one or more OH groups in the whole molecule. Fucols consist of phloroglucinol units linked by aryl-aryl bonds, and fucophlorethols of phloroglucinol units linked through a mixture of ether and phenyl bonds. Eckols and carmalols are characterized by the presence of a dibenzodioxin unit; eckols differ from carmalols by their usually low molecular weight and by the presence of a phenoxyl substitution at C4 [23,24].

Besides their structural, chemical and biological functions in brown seaweeds, phlorotannins have been recognized over the years as promising bioactive compounds with potential health benefits in a wide variety of human diseases [22]. Among them, the capacity of these polyphenols to prevent the onset and to slow down the progression of DM, as well as to treat diabetes-related complications, has began to be explored in the recent few years.

### 3.1. Inhibition of Glucose Absorption

**Figure 3:**
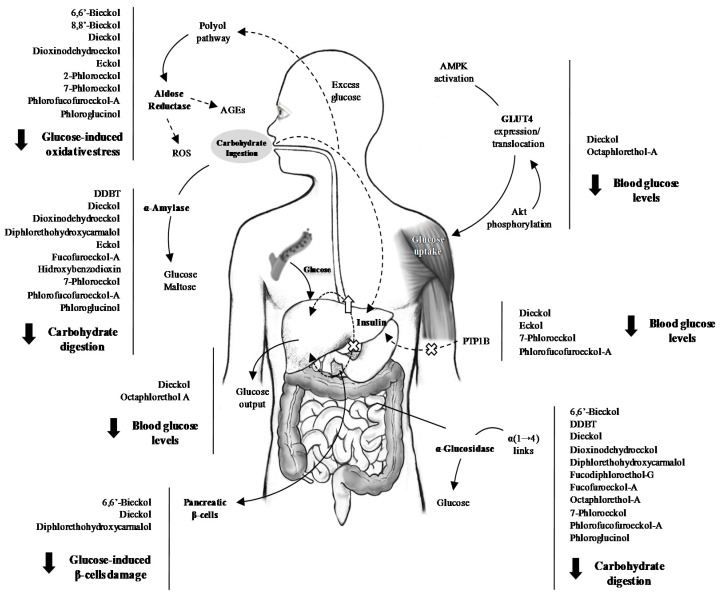
Schematic representation of the main diabetes-related targets of phlorotannins (AGEs: advanced glycation end-products; Akt: protein kinase B; AMPK: AMP-activated protein kinase; DDBT: 2-(4-(3,5-dihydroxyphenoxy)-3,5-dihydroxyphenoxy) benzene-1,3,5-triol; G6P: glucose-6-phosphatase; GLUT4: glucose transporter 4; PEPCK: phosphoenolpyruvatecarboxykinase; PTP1B: protein tyrosine phosphatase 1B; ROS: reactive oxygen species).

Cleavage of complex carbohydrates from diet is essential for their absorption in the small intestine. α-Glucosidase and α-amylase are the main gastrointestinal enzymes responsible for cleaving dietary carbohydrates (Figure 3).

α-Amylase is predominant in saliva and pancreatic juice, and is responsible for the hydrolysis of the α-bonds of complex carbohydrates (starch and glycogen) to give glucose and maltose. Its role in carbohydrate cleavage is supplemented by the action of α-glucosidase, present in the brush border of the small intestine, and responsible for the hydrolysis of α-(1→4) links, releasing glucose for enterocyte uptake. Drugs able to inhibit the activity of α-glucosidase and α-amylase can retard the digestion of carbohydrates, thus reducing postprandial hyperglycaemia [25]. Three pharmaceutical formulations are based on molecules able to slow down carbohydrates assimilation through the blockage of digestive enzymes: miglitol and voglibose are both α-glucosidase inhibitors, whereas acarbose is a dual α-glucosidase and α-amylase inhibitor [26,27,28]. α-Glucosidase inhibitors act by competitive inhibition of the active site of the enzyme, preventing the cleavage of carbohydrates and delaying glucose absorption. Despite the efficacy of the marketed molecules in reducing postprandial glycaemia, they are still expensive, are contraindicated in chronic intestinal disease and require a regular monitoring of hepatic transaminases. The adverse gastrointestinal effects of α-glucosidase inhibitors, like flatulence, abdominal discomfort and, sometimes, diarrhoea are the main responsible for therapy discontinuation [6].

A wide diversity of brown seaweeds extracts and isolated phlorotannins have been explored for their ability to suppress carbohydrate digestion and glucose absorption (Figure 3). Of the species studied in the last decade, *Ecklonia* sp. and *Eisenia* sp. are, by far, the most widely explored in this respect (Table 1).

**Figure 4:**
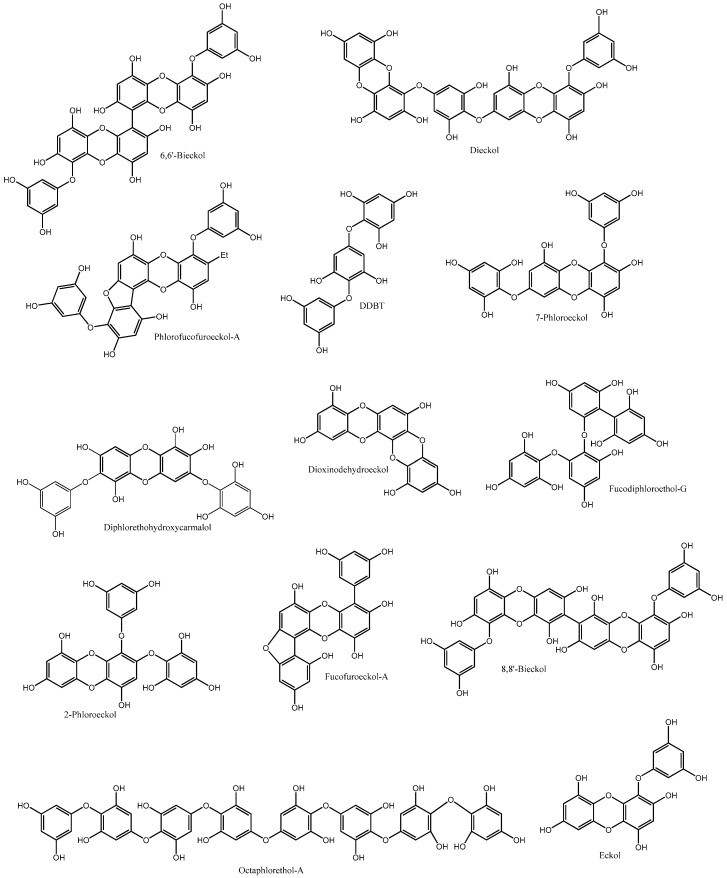
Chemical structures of phlorotannins with anti-diabetic activity isolated from brown seaweeds.

Lee et al. [29] isolated five phlorotannins from *Ecklonia cava* (fucodiphloroethol-G, dieckol, 6,6′-bieckol, 7-phloroeckol and phlorofucofuroeckol-A (PFF-A), Figure 4), and evaluated their capacity to inhibit rat intestinal α-glucosidase and porcine pancreatic α-amylase. All the isolated phlorotannins were active against α-glucosidase (10.79 < IC_50_ < 49.49 µM), presenting a weaker activity against the pancreatic enzyme (IC_50_ > 124 µM). The inhibitory activity was dose dependent, dieckol being the most effective compound against both enzymes (IC_50_ = 10.79 and 124.98 µM for α-glucosidase and α-amylase, respectively). The authors approached the inhibition pattern of dieckol and concluded that it acted through a non-competitive mechanism. Unlike most α-glucosidase inhibitors, which act by mimicking the pyranosyl moiety of α-glucosidase, phlorotannins may form complexes with the enzymes, for which the OH groups are mandatory.

Moon et al. [34] explored the genus *Ecklonia* and *Eisenia*, from which six bioactive compounds were isolated: phloroglucinol, dioxinodehydroeckol (DDE), eckol, PFF-A, dieckol and 7-phloroeckol (Figure 4). Phlorofucofuroeckol-A (IC_50_ = 1.37 µM), followed by dieckol (IC_50_ = 1.61 µM), were the most efficient compounds against α-glucosidase, being stronger than the reference drug, acarbose. Interestingly, and contrary to what happened in the study undertaken by Lee et al. [29], these authors reported that dieckol acted competitively with acarbose [34].

The explanation for the succeeded may lay in the methodology used for determining the kind of inhibition, as Moon et al. used the Dixon plot method, known for its limitations in distinguishing between competitive and non-competitive enzyme inhibition [50]. Dieckol was further explored by another research group [35]. The IC_50_ of the compound, isolated from the same species, was found to be lower than that of the reference drug, for both α-glucosidase (0.24 face to 1.05 mM) and α-amylase (0.66 face to 1.09 mM). Moreover, it was not cytotoxic for human vein endothelial cells (HUVEC) and promoted a suppression of postprandial blood glucose levels.

Eckol, dieckol, and 7-phloroeckol, isolated from *Eisenia bicyclis*, presented α-amylase inhibitory capacity of more than 87%, at a concentration of 1 mM. The inhibitory capacity of the isolated compounds was positively correlated with their molecular weight, as well as with the number of free OH groups [33]. Fucofuroeckol-A (FF-A) (Figure 4) and DDE were later isolated from the same species, and evaluated for their capacity to inhibit carbohydrate digesting enzymes [40]. Both compounds demonstrated significant inhibitory activities against both enzymes: FF-A presented an IC_50_ of 131.34 nM for α-glucosidase and of 42.91 µM for α-amylase, while the values presented by DDE were 93.33 nM and 472.7 µM, respectively. Both isolated compounds were more effective against α-glucosidase than acarbose (IC_50_ = 51.65 µM) [40]. Diphlorethohydroxycarmalol (DPHC) (Figure 3), isolated from *Ishige okamurae* [41], and DDBT (Figure 4), isolated from *Sargassum patens* [32], are other examples of non-toxic phlorotannins with α-glucosidase and α-amylase inhibitory capacity. The kinetic study additionally demonstrated that DDBT inhibited α-amylase (IC_50_ = 3.2 µg/mL) by competition with the substrate [32]. In addition to isolated compounds, the ability of phlorotannin extracts to inhibit carbohydrates digesting enzymes has also been explored (Table 2).

A 75:25 *v*/*v* water-methanol extract of *Padina pavonica* displayed activity against both α-glucosidase (IC_50_ = 34.40 mg/mL) and α-amylase (IC_50_ = 0.25 mg/mL) in a dose dependent manner, though not as strong as that of acarbose (IC_50_ = 1.74 and 0.06 mg/mL, respectively). The IC_50_ found for the forming unit, phloroglucinol was higher than that of the extract for both enzymes [48]. Cold water and ethanol extracts of *Ascophyllum nodosum* and *Fucus spiralis* strongly inhibited α-amylase and α-glucosidase bellow cytotoxic concentrations. *A. nodosum* methanolic extract was more potent against α-amylase (IC_50_ = 44.7 µg/mL), while *F. spiralis* demonstrated more activity against α-glucosidase (IC_50_ = 0.32 µg/mL). The activity was positively correlated with the phenolic content and antioxidant activity of the extracts [60]. A methanolic extract of *Spatoglossum asperum* displayed inhibitory activity for both enzymes, with an IC_50_ similar to those found for acarbose [57].

It seems obvious that the solvent used in the extraction process influences the bioactivity, not only because it affects the amount of phenolic compounds extracted, but also because of the different phlorotannins classes, with different molecular weights and degree of hydroxylation, that will predominate according to the polarity of the solvent [23]. For instance, Eom et al. [40] tested the activity of isolated phlorotannins and of different extracts against the carbohydrate digesting enzymes. The ethyl acetate fraction, which presented the highest phenolic content, was also the one displaying the lowest IC_50_ values for both enzymes. On the other hand, the dichloromethane fraction, showing higher content of phenolic compounds than the *n*-butanol one, demonstrated a lower ability to inhibit carbohydrate-digesting enzymes. Thus, it can be assumed that the phlorotannins present in the *n*-butanol fraction, even at lower amounts, may belong to different classes than those extracted with dichloromethane, presenting higher activity. In a similar study, Pantidos et al. [59] tested different extracts of the edible seaweed *A. nodosum*. The polyphenolic extract of this seaweed was more active than the tannins-rich fraction against α-amylase, while no differences were observed for α-glucosidase, again suggesting that the inhibition of these enzymes is strongly influenced by polyphenol classes. The authors verified that the tannins-rich fraction inhibited both enzymes and presented a synergistic effect with acarbose, which emphasizes the idea that phlorotannins do not only cause inhibition by acting at the enzymes’ catalytic centre [59]. 

Faced with the above, it seems obvious that phlorotannins are potent candidates for inhibiting digestive enzymes, considering their efficacy, low effective doses and reduced cytotoxicity. However, much controversy exists in what concerns their effective doses and mechanism of action. On the one hand, because of the different methodologies followed by the diverse research groups, and on the other because of the enzymes’ origins and the mathematical analyses themselves, the absolute IC_50_ values for isolated phlorotannins, and even for the reference drugs, widely vary, emphasizing the need for protocol standardization. Nevertheless, the ability of phlorotannins to combat diabetes-associated factors is so recognized that a phlorotannins-based commercial preparation is already being marketed [61]. InSea2™ consists in a hot water extract containing phlorotannins derived from the species *A. nodosum* and *Fucus vesiculosus.* This formula inhibits both α-amylase and α-glucosidase, and has induced a reduction of 48% in blood glucose levels, 12% in insulin secretion and 8% improvement in insulin sensitivity, with a safe profile and without gastrointestinal upset [61]. IC_50_ values of 2.8 and 5.0 µg/mL were found for α-amylase and α-glucosidase, respectively and, considering its overall profile, it was indicated as suitable for use in food and dietary supplements [62].

### 3.2. Effects on Postprandial Hyperglycaemia and Insulin Levels In Vivo

Evidence suggests that postprandial hyperglycaemia constitutes an independent risk factor for cardiovascular complications in both diabetic and non-diabetic individuals, the loss of its control being the first step in the deterioration of glucose homeostasis [63]. The postprandial phase is characterized by a rapid and large increase of blood glucose levels that originate postprandial hyperglycaemic spikes [15]. The increase of blood glucose levels and of released insulin will in turn inhibit glucagon secretion and release, promoting glucose uptake in various tissues. However, in diabetic individuals, insulin resistance leads to a lower reduction of glucagon secretion by the pancreas and, thus, to an inappropriate glucose production and to an inefficient glucose uptake, which results in increased postprandial hyperglycaemia and prolonged elevation of plasma glucose [63]. As postprandial hyperglycaemia is associated with carotid thickening and used to predict infarction, its control may mitigate cardiovascular risks, which are the main causes of morbidity and mortality in diabetic individuals [15,64].

The effect of phlorotannins on postprandial glycaemia levels has been achieved using streptozotocin (STZ)-induced diabetic mice models. Of the phlorotannins known heretofore, dieckol has probably been the most extensively studied [35,36,39,51,65] (Table 1). Lee et al. [35] isolated dieckol from *E. cava* and evaluated the effect of its oral administration in alleviating the postprandial hyperglycaemia of STZ-induced diabetic mice. The increase of postprandial blood glucose levels was significantly suppressed in both normal (AUC = 200 mmol·min/L) and diabetic animals (259 mmol·min/L) fed after dieckol administration, face to the respective controls (AUC= 374 and 483 mmol·min/L). The same compound was later evaluated in a type II diabetic mouse model (C57BL/KsJ-*db*/*db*), through intraperitoneal injection of dieckol instead of oral administration, at doses of 10 and 20 mg/kg [36]. The dieckol-treated group presented significantly lower blood glucose and insulin levels compared to the control. In a recent survey, Lee et al. [51] evaluated the effect of a dietary supplementation with a dieckol-rich extract from *E. cava* (1500 mg/day) versus placebo in several biological parameters of pre-diabetic human adults. The randomized, double-blind, placebo-controlled trial was carried out during 12 weeks, after which the dieckol-rich extract treated group showed a significant decrease of postprandial glucose levels. No significant adverse events related to the consumption of the dieckol-rich extract were reported. Furthermore, a significant decrease in insulin levels was reported, suggesting a reduction in insulin resistance.

Other phlorotannins seem promising in reducing blood glucose levels (Table 1). Diphlorethohydroxycarmalol isolated from *I. okamurae* significantly suppressed the increase of postprandial glucose levels in STZ-induced diabetic mice. After overnight fasting, mice were administered orally soluble starch, alone or with DPHC or acarbose. The AUC of postprandial glucose levels of mice fed the phlorotannin was significant reduced face to control (2022 face to 2210 mmol·min/L) and similar to those of acarbose (1964 mmol·min/L) [41]. Recently, PFF-A isolated from *E. cava* demonstrated ability to alleviate postprandial hyperglycaemia caused by starch [47]. Increases in postprandial blood glucose levels were significantly more suppressed in groups administered PFF-A when compared to control, for both normal and diabetic mice. Similarly to what happened with DPHC, the AUC of postprandial glucose levels of diabetic mice fed the phlorotannin was significant reduced face to control (2296 vs. 2690 mmol·min/L) and similar to those of acarbose (2231 mmol·min/L).

Besides the isolated compounds, the ability of phlorotannin extracts to alleviate postprandial hyperglycaemia has also been explored (Table 2). Lee et al. [55] evaluated the effect of a phlorotannins rich extract of *Sargassum ringgoldianum* on carbohydrate digesting enzymes and on postprandial hyperglycaemia in STZ-induced diabetic mice. Two hours after starch ingestion, animals treated with phlorotannins (oral administration) presented a significant decrease in blood glucose levels (307 mg/dL) when compared to the untreated control group (403.5 mg/dL). Additionally, blood glucose levels of both normal and diabetic animals treated with phlorotannins and acarbose were similar. This hypoglycaemic effect was attributed to the inhibition of carbohydrate digesting enzymes, which can delay the absorption of dietary carbohydrates in the intestine, leading to the suppression of glucose levels after a meal [55]. Other surveys with diabetic mice proved the efficacy of phlorotannins in alleviating postprandial hyperglycaemia. Diets supplemented with polyphenol-rich extracts of *I. okamurae* resulted in the reduction of plasma glucose levels, also improving insulin resistance in type 2 *db*/*db* diabetic mice [58]. The same effect was reported for a dieckol-rich extract of *E. cava*, which significantly improved blood glucose levels face to control, and impaired glucose tolerance in type 2 diabetic mice following supplementation for 6 weeks [52]. The potential of phlorotannins to reduce postprandial glucose levels has also been demonstrated by the commercial formulation InSea2™, able to reduce the normal increase in postprandial blood glucose by 90%, 30 min after a meal, and consecutively reducing the peak insulin secretion by 40% [62].

It is a fact that the inhibition of the pancreatic α-amylase and the intestinal α-glucosidase represent one of the first lines of action in the prevention of postprandial blood glucose levels rises in diabetic individuals. Considering the evidence provided by phlorotannins in reducing postprandial glucose, it seems evident that a great part of their activity is related to their capacity to inhibit glucose absorption through the inhibition of carbohydrate-digesting enzymes.

### 3.3. Endogenous Glucose Production

Besides dietary carbohydrates, fasting hyperglycaemia is largely the result of glucose overproduction by the liver, one of the primordial organs in the control of glucose homeostasis (Figure 3). Hepatic alterations can have a drastic influence on glucose homeostasis, representing the first sign of DMT2. Two main enzymes are responsible for balancing the hepatic glucose production: glucokinase and glucose-6-phosfatase (G6P) [66]. Glucokinase plays a major role in controlling glucose phosphorylation and metabolism, both in liver and in pancreas. Its activity is increased by insulin, produced by pancreatic β-cells in response to increased blood glucose levels, resulting in an enhanced uptake of blood glucose by the liver. On the other hand, G6P is a key enzyme in the control of hepatic gluconeogenesis and glucose hepatic output, being suppressed by the action of insulin. In diabetic individuals, the activity of glucokinase is markedly decreased, while that of G6P is enhanced [67]. Thus, these enzymes can be seen as therapeutic targets in diabetic individuals, and the modulation of their activity a mean of controlling blood glucose levels, thus avoiding diabetes-related complications.

There are few studies exploring the effect of phlorotannins on glucokinase and G6P activity. However, those available highlight the potential of these polyphenols in controlling hyperglycaemia (Table 1 and Table 2). Lee et al. explored the effect of a dieckol-rich extract on diabetic mice and observed that the glucokinase activity was significantly higher in mice fed a diet supplemented with dieckol extract, while G6P activity was significantly suppressed, resulting in a decline in hepatic glucose production, similar to what happened in the presence of a glucose sensitizer [52]. A previous study with a phlorotannins-rich extract has already demonstrated the ability of these compounds to enhance glucokinase activity, though no influence in G6P had been reported [58]. Among glucose regulating genes, those encoding for G6P and phosphoenolpyruvate carboxykinase (PEPCK) have been found over-expressed in most forms of diabetes. These rate limiting enzymes in the gluconeogenesis process contribute to an increased hepatic glucose output, being inhibited by insulin [68]. In a recent survey, Lee et al. [46] explored an isolated phlorotannin, octaphlorethol-A (OPA) (Figure 4), in diabetes-related mechanisms. Among others, the isolated compound significantly suppressed the increase in hepatic mRNA expression levels of G6P and PEPCK, indicating that OPA can effectively contribute to the suppression of hepatic glucose output. In fact, these observations allow us to classify phlorotannins as multi-target antidiabetic agents once, if in the one hand they have the ability to reduce the absorption of carbohydrates from diet, reducing postprandial hyperglycaemia, on the other hand, they act on hepatic enzymes, reducing endogenous glucose production and contributing to the control of fasting hyperglycaemia.

### 3.4. Aldose Reductase and the Polyol Pathway

Aldose reductase (AR) has a marked position in the pathogenesis of DM. It constitutes a crucial rate-controlling enzyme in the polyol pathway and is implicated in the pathogenesis of various diabetes-related vascular complications [69]. Briefly, under hyperglycaemic conditions, a significant amount of non-phosphorylated glucose enters the polyol pathway, being reduced to sorbitol by AR, at the expense of NADPH. However, since NADPH is essential for the generation of the endogenous antioxidant glutathione (GSH), its depletion through the AR pathway can compromise the intracellular antioxidant defences. In a second step, sorbitol is converted to fructose by sorbitol dehydrogenase, with the reduction of NAD+ to NADH, a substrate of NADH oxidase that leads to the production of superoxide anions. In a third step, fructose itself can be converted to fructose-3-phosphate and 3-deoxyglucosone, which constitutes a potent glycation agent, leading to the formation of reactive oxygen species (ROS) through advanced glycation end-products (AGEs). The mechanisms involving the polyol pathway are considered one of the most important for the development of DM complications, mediating metabolic and osmotic alterations in many tissues [70]. Indeed, the inhibition of the polyol pathway seems to be such an important strategy to prevent diabetes complications like cardiomyopathy, neuropathy, nephropathy and retinopathy, that a number of AR inhibitors are being investigated [71,72].

Although phlorotannin exploitation for AR inhibition is still scarce, the existing studies reveal potency comparable to that of well-known AR inhibitors [30,37,73] (Table 1, Figure 3). The effect of phlorotannins was evaluated in an AR-rich homogenate from rat lens [37]. The ethyl acetate fraction of *Ecklonia stolonifera* was the most effective in inhibiting AR and, of the isolated phlorotannins, DDE presented the lowest IC_50_ (21.95 and 27.54 µM, respectively). Interestingly, 7-phloroeckol, the second most active compound, presented an IC_50_ (27.54 µM) significantly lower than that of 2-phloroeckol (99.62 µM) (Figure 3), which may presume that the inhibitory activity strictly depends on the position of the additional phloroglucinol unit in the dibenzodioxin nucleus (Figure 4). In another survey [30], several phlorotannins isolated from *E. bicyclis* were evaluated for their capacity to inhibit a recombinant human aldo-keto reductase (rhAKR1B10), and their bioactivity was compared to that of epalrestat used as reference. The inhibitory rate of PFF-A was 61.41% at 10 µM, with an IC_50_ of 6.22 µM, and the enzyme kinetic analyses revealed non-competitive inhibition. Although 50% inhibition could not be reached by dieckol, 6,6′-bieckol and 8,8′-bieckol (Figure 4), the percentages of AR inhibition presented by these compounds (10 µM) were significantly variable (27.34%, 9.86% and 48.55%, respectively), highlighting the importance of the position of phloroglucinol units within the molecules.

In part because of their toxicity, several AR inhibitors have failed Phase III clinical trials, or have been discontinued early. Despite numerous efforts made over the last few decades, epalrestat is the only commercially available AR inhibitor to date, being marketed only in India and Japan [71]. Although there are few studies regarding the effect of phlorotannins on AR, their bioactivity seems undeniable. Additionally, the absence of toxicity of these polyphenols, previously reported on several cell lines [35,74], may be a step ahead regarding the development of new, efficient and safe AR inhibitors. 

### 3.5. Glucose Uptake

#### 3.5.1. Protein Tyrosine Phosphatase 1B (PTP1B)

Protein-tyrosine phosphatase 1B (PTP1B) is a negative regulator of insulin signalling, located in the endoplasmic reticulum membrane of hepatic, muscular and adipose tissues (Figure 3). Studies on the cellular role of PTP1B have clearly shown that this enzyme acts as a key negative regulator of the tyrosine phosphorylation cascade, which is an integral part of the insulin signalling pathway [75]. Insulin receptor (IR) belongs to the class of tyrosine kinase receptors. Binding of insulin leads to structural changes in the IR that results in the phosphorylation of several tyrosine residues within the cell and, consequently, to the activation of different pathways that culminate with an increased glucose uptake. Due to its ubiquity in the insulin-targeted tissues and its reported role in insulin resistance development, PTP1B is considered a promising therapeutic target for the treatment of DMT2 [75,76]. The unique survey concerning to the effect of phlorotannins on IR phosphorylation has demonstrated promising perspectives. Moon et al. [34] evaluated the effect of phlorotannins isolated from *E. cava* and *E. bicyclis* in human recombinant PTP1B. Eckol, PFF-A, dieckol and 7-phloroeckol were potent and non-competitive PTP1B inhibitors, IC_50_ values ranging from 0.56 to 2.64 µM. Face to the results, and in a structure-activity relationship attempt, the authors considered that a relative large molecular size and number of OH groups would be key determinants for PTP1B inhibition.

#### 3.5.2. Glucose Transporter 4 (GLUT4)

Glucose uptake by muscle cells constitutes a critical step for reducing blood glucose levels [77]. Besides being the primary tissue for glucose uptake and disposal, muscle is also an important target regarding DMT2 [78]. In muscle cells, insulin stimulates glucose uptake via translocation of vesicles containing facilitate carrier proteins (GLUT4), which are highly expressed in this tissue. The translocation of GLUT4 is, in turn, dependent on the activation of AMP-activated protein kinase (AMPK), which plays a major role in energy homeostasis in ATP-depleting metabolic states. Under conditions of ischemia, hypoxia, heart shock and oxidative stress, AMPK induces ATP generation through glucose uptake, by regulating GLUT4 expression [77].

The up-regulation of GLUT4 is related to enhanced glucose transport and utilization by muscle and, consequently, to the decrease of blood glucose levels (Figure 3). Octaphlorethol A has recently been the subject of intensive studies related to diabetes condition [43,44,45,46] (Table 1). Type 2 diabetic mice were intraperitoneally daily injected with OPA for five weeks. Besides the lower glucose levels, OPA treated group presented a significant reduction of serum insulin levels (2.51 ng/mL) when compared to control (4.81 ng/mL). The increased AMPK activation observed in OPA group resulted in an enhanced expression of GLUT4 in skeletal muscle cells and, consequently, in a significant increase in glucose uptake by muscle. The available studies suggest that the AMPK signalling pathway is responsible for the up-regulation of GLUT4 expression in diabetic mice after OPA treatment, which results in enhanced glucose uptake and lower blood glucose levels [43,46]. The effect of dieckol, isolated from *E. cava*, in the glucose uptake by muscle cells has recently been evaluated, using zebrafish (*Danio rerio*) as a model of human disease [79]. Besides the decrease in blood glucose levels and the reduced expression of G6P and PEPCK observed in fish treated with dieckol, an increased phosphorylation of protein kinase B (Akt) was observed in *D. rerio* muscle tissue. Akt is an important signalling molecule, required to induce glucose transport through the insulin-induced translocation of GLUT4 to the plasma membrane [80]. Akt activation was involved in mediating the effect of dieckol on glucose transport activation and insulin sensitivity. The authors proved that the strong anti-diabetic effect of dieckol was exerted by improving blood glucose regulation, hepatic glucose metabolic regulation and Akt up-regulation in diabetes-induced zebrafish. Further studies with dieckol isolated from *E. cava* demonstrated that dietary supplementation with this phlorotannin lead to the reduction of C-peptide, a biomarker of insulin secretion, and to a two-fold increase in plasma adiponectin levels in diabetic mice, compared with the untreated control group [81]. The compound was pointed as an activator of the insulin signalling pathway because of its capacity to increase the phosphorylation of IR, PI3 kinase and Akt. GLUT4 was up-regulated in dieckol treated mice, suggesting that the isolated phlorotannin controls plasma glucose levels by interference with the expression of GLUT4. In fact, the anti-diabetic therapeutic effect of *E. cava* extracts had already been demonstrated [82]. Besides reducing the symptoms of polyphagia and polydipsia, characteristics of the disease, the methanolic extract of this brown seaweed also activated the AMPK, PI3 kinase and Akt signalling pathways.

### 3.6. Glucose-Induced Toxicity

#### 3.6.1. AGEs

The deleterious effects attributable to hyperglycaemia make it the principal cause of diabetic complications. Thus, the increased availability of glucose in diabetic individuals leads to a markedly accelerated formation of AGEs. AGEs constitute a heterogeneous group of molecules formed from non-enzymatic reaction of reducing sugars with free amino groups of carrier proteins in blood vessels, structural proteins, enzymes, lipids and nucleic acids [83]. Methylglyoxal, a metabolite of glucose, is the most reactive AGE precursor. This highly reactive dicarbonyl compound is spontaneously formed during glycolysis and reacts with proteins to irreversibly yield AGEs [84]. These compounds can alter proteins structure and function, affecting cellular matrix, membranes and vessel-wall components; they interact with AGE receptors in the cell surface, leading to their endocytosis and degradation, and to the activation of pro-oxidant and pro-inflammatory events. Thus, AGEs are doubtlessly one of the most important pathogenic mediators of almost all diabetes complications, being found in many different tissues of diabetic individuals, and positively correlated with the severity of the lesions [83,85]. Glycation inhibition has been shown to retard the development of diabetic complications and consequently, anti-AGE drugs are being extensively studied. The first drug designed to inhibit glycation reactions was aminoguanidine [83]. It inhibits the conversion of early products to AGEs and has proved its benefits in many diabetes-related complications. Drugs able to disrupt already formed AGEs or to prevent their formation, are of such importance in reducing diabetes complications, that active research has developed in this field [21,83,85].

Some approaches regarding the capacity of phlorotannins to avoid diabetes-related complications had been made (Table 1). Okada et al. [33] isolated three phlorotannins from *E. bicyclis* (eckol, dieckol and 7-phloroeckol) and assessed their ability to inhibit proteins glycation by ELISA. The percentages of glycation inhibition were higher than 85% for all of the isolated compounds (1 mM), and also higher than that of the reference compound aminoguanidine. An inhibition of almost 100% was achieved by eckol (1 mM) [33]. Jung et al. [37] evaluated the capacity of different fractions of an ethanolic extract of *E. stolonifera* to inhibit AGEs formation. When compared to the positive control aminoguanidine, the ethyl acetate fraction was the most active (46.6% inhibition), and thus subjected to column chromatography to yield phloroglucinol, DDE, eckol, PFF-A, dieckol, triphlorethol-B, 2-phloroeckol and 7-phloroeckol. Of the isolated compounds, PFF-A displayed significant AGEs inhibitory effect (IC_50_ = 165.20 mM), compared to aminoguanidine (IC_50_ = 291.45 mM). Heo et al. [86] isolated DPHC from *I. okamurae* and evaluated its effect on glucose-induced cytotoxicity in HUVEC. DPHC was shown to prevent cells from high glucose-induced damage, restoring cells viability. The levels of intracellular ROS and reactive nitrogen species (RNS) was also significantly reduced by DPHC, demonstrating the ability of this phlorotannin to reduce the damage caused by high glucose-induced oxidative stress (GIOS) associated with diabetes [86]. The same research group also evaluated the effect of dieckol [38] and 6,6′-bieckol [31] isolated from *E. cava* on GIOS, using the same cell line. The authors verified that both compounds significantly inhibited cell death induced by high glucose treatment (30 mM), at concentrations of 10–50 µg/mL. Moreover, both compounds reduced the glucose-induced over-expression of inducible nitric oxide synthase, cyclooxygenase-2 and nuclear factor-kappa B proteins in HUVEC.

In addition to isolated phlorotannins, some surveys have explored the capacity of phlorotannin-rich extracts to prevent glucose-induced toxicity (Table 2). Liu et al. [49] observed that phlorotannins-rich extracts from *Fucus vesiculosus* significantly inhibited the formation of AGEs mediated by glucose and methylglyoxal, in a dose-dependent manner. Phlorotannins-rich extracts were capable of inhibiting bovine serum albumin (BSA) glycation, more than or as effectively as aminoguanidine. The authors proceeded with phlorotannins fractionation and chemical characterization by HPLC-MS^n^, and verified that those of lower molecular weight were more effective in inhibiting protein glycation mediated by glucose. Phloroglucinol, the forming unit of phlorotannins, was the most effective anti-glycation agent (EC_50_ = 0.058 mg/mL face to 0.197 mg/mL of aminoguanidine). The authors demonstrated that phlorotannins were able to scavenge reactive carbonyls through the formation of adducts, this being the major mechanism for protein glycation inhibition [49]. Recently, Shakambari et al. [56] explored the protective effect of phlorotannins extracts from *P. pavonica*, *Sargassum polycystum* and *Turbinaria ornata* against AGEs formation in vitro, through the BSA-glucose assay. Contrary to the previous study, the IC_50_ of the extracted phlorotannins was found to be lower (15–32 µg/mL) than that of phloroglucinol (222 µg/mL), used as standard. Phlorotannins from the three brown seaweeds also exhibited protective effects against AGE formation in vivo in hyperglycaemia-induced *Caenorhabditis elegans*, an animal model used to study glucose toxicity. 

#### 3.6.2. Protection of Pancreatic β-Cells

It is well known that chronic oxidative stress induced by enhanced hyperglycaemia constitutes a central mechanism for glucose toxicity in pancreatic β-cells. [87]. Endogenous ROS contribute to maintain organism homeostasis; however, its accumulation for prolonged periods can lead to chronic oxidative stress. ROS can directly damage lipids, proteins or DNA, as well as modulate intracellular signalling pathways, causing changes in protein expression and, therefore, irreversible oxidative modifications. This is particularly harmful for pancreatic islet cells, once they are among the tissues with the lowest levels of intrinsic antioxidant defences. Among those, the endogenous enzymes catalase (CAT), superoxide dismutase (SOD) and glutathione peroxidase (GPx) stand out [88,89]. The glucotoxic state results in deleterious consequences for the β-cells function: chronic exposure to supra-physiologic glucose concentrations can cause defective insulin gene expression, leading to a decline in insulin production and to its abnormal secretion [90]. For instance, in hyperglycaemia-dependent lipotoxicity, the high glucose-induced production of reactive species increases lipid peroxidation, making the prolonged exposure of pancreatic β-cells to fatty acids affect insulin gene expression and insulin secretion; under high glucose concentrations, elevated fatty acids are not promptly oxidized in mitochondria, being diverted towards different pathways that result in the production of deleterious products [91]. These lipid peroxides, thiobarbituric acid reactive substances (TBARS), are significantly increased in diabetic patients, resulting in a progressive deterioration of β-cells function [92]. In more severe situations, hyperglycaemia can lead to the apoptosis of β-cells [93,94].

As it is unquestionable that continuous high glucose levels are deleterious for pancreatic β-cells, a question has arised: is the increment in antioxidant host defences an appropriate therapeutic strategy to reduce the impact of diabetes and hyperglycaemia on β-cells? In fact, some authors have questioned the role of phlorotannins in this regard. The protective effect of 6,6′-bieckol purified from *E. cava* was recently investigated against high glucose-induced toxicity using rat insulinoma cells [95]. The authors observed that treatment with 6,6′-bieckol dose-dependently reduced the levels of TBARS, intracellular ROS and nitric oxide, which levels had been increased by the high glucose concentration (30 mM). Cells protection from apoptosis was also reported for 6,6′-bieckol treatment; 50 µg/mL of the isolated compound had a protective effect against cellular damage induced by 30 mM glucose, resulting in a significant increase in cell viability (70%) face to control (33%). A further analysis revealed that 6,6′-bieckol markedly reduced the expression levels of the pro-apoptotic protein Bax, which is highly expressed under high glucose concentrations, and increased the expression of the anti-apoptotic protein Bcl-2 [95]. The protective effect of 6,6′-bieckol against reactive species had been previously assessed by the same research group on HUVEC subjected to hyperglycaemic conditions, pointing it as a potential therapeutic agent in the prevention of diabetic endothelial dysfunction and related complications [31]. 

Several surveys have demonstrated that dieckol, found in high amounts in *E. cava*, is a promising candidate for the prevention and treatment of diabetes-related complications [35,36,38,39,51,79,81] (Table 1). Its protective effect against β-cells death through GIOS was demonstrated by Lee et al. [39]. Dieckol significantly inhibited glucose-induced toxicity at a concentration of 17.5 µM, through the reduction of TBARS and of the generation of intracellular ROS and RNS. Moreover, this phlorotannin increased the activity of antioxidant enzymes, such as CAT, SOD and GPx, in high-glucose pre-treated insulinoma cells. As it happened with 6,6′-bieckol, dieckol affected the expression of anti-apoptotic and pro-apoptotic proteins, increasing the expression of Bcl-2 and reducing that of caspase-3 [39]. Beyond its beneficial effect on GIOS on pancreatic β-cells, dieckol has also demonstrated in vivo hepatoprotective effect on ethanol induced hepatic damage in BALB/c mice [65]. This phlorotannin lowered hepatic transaminases and SOD levels and promoted a reduction in total cholesterol.

Besides *Ecklonia*, the genus *Ishige* seems promising regarding phlorotannins with anti-diabetic potential [41,42,43,44,45,58,86] (Table 1). OPA (Figure 4) isolated from *Ishige foliaceae* significantly reduced cell death in STZ-treated pancreatic β-cells. STZ acts as a potent DNA-methylation agent, able to generate ROS, to increase lipid peroxidation and to induce oxidative stress in pancreatic β-cells [96]. OPA treatment reduced TBARS and stimulated the activity of the antioxidant enzymes CAT, SOD and GPx, at the lowest concentration tested (12.5 µg/mL) [44]. Its effect was also reflected in insulin levels: a 24 h treatment of pancreatic β-cells with STZ significantly decreased insulin levels; however, treatment with 50 µg/mL of OPA lead to a significant increase in insulin levels (from 1.36 to 3.85 ng/mL). The flow cytometry analysis revealed that a 50 µg/mL OPA pre-treatment resulted in a significantly reduction in cell apoptosis. This can be attributed to the capacity of OPA to markedly increase the expression of the anti-apoptotic protein Bcl-xl and to reduce the expression of cleaved caspase-3 in STZ-treated cells [44].

The antioxidant capacity of DPHC, a carmalol isolated from *I. okamurae*, was evaluated in RINm5F pancreatic β-cells subjected to a high glucose environment (30 mM) [42]. DPHC significantly inhibited glucotoxicity and apoptosis, reducing TBARS formation in a dose-dependent manner, and increasing the activity of CAT, SOD and GPx in high glucose pre-treated RINm5F cells. The isolated compound also promoted cell survival and increased insulin secretion by pancreatic β-cells, which had been inhibited by the high glucose concentration. Altogether, the available studies point phlorotannins as potential pharmaceutical agents or nutraceuticals, capable of preventing β-cells destruction caused by GIOS, common in diabetic individuals.

## 4. Study Perspectives and Conclusions

Several biological activities have been attributed to phlorotannins over the years. From their marked antioxidant potential, typical of polyphenols and intrinsically related to the pathogenesis of diverse diseases, to their capacity of influencing gene expression, the range of activity of phlorotannins is indeed remarkable. Studies on the anti-diabetic activity of these compounds, explored in depth in recent years, have confirmed what was already known among the scientific community: their biological potential and broad mechanism of action. Actually, the surveys explored throughout this review are an incontestable proof of that. Phlorotannins are not only promising in avoiding hyperglycaemia, through their extraordinary capacity to inhibit glucose absorption, but are also capable of treating diabetes-associated disorders through their capacity of protecting β-cells from glucose-induced toxicity, in diagnosed DMT2 individuals. To date, the in vivo studies available are a clear evidence of phlorotannins capacity to invert a situation of cell death triggered by high glucose levels.

While these polyphenols seem to constitute a pharmacological approach to control hyperglycaemia and to treat diabetes-associated vascular complications, without providing toxicity, there is still a long way to go. For instance, most of the available studies are conducted on non-human enzymes isolates, making it difficult to extrapolate results, not only because of the different enzymatic catalytic centres, but also because of the coexisting complex biological environment of living organisms, increasing the possibility of treatment failure. The lack of standard protocols, their degree of reproducibility and the correlation between in vitro and in vivo data, hinders stronger assumptions regarding the therapeutic potential, suitability and safety of new compounds. In addition to the referred issues, expected at the beginning of new molecules investigation, major concerns regarding seaweed bioavailability stand out. In fact, at the moment, phlorotannins are not chemically biosynthesised, and the amounts of raw material needed for their isolation, as well as the associated costs of the procedure, makes their utilization as anti-diabetic medicines, at least for now, completely unfeasible. Nevertheless, considering their proved biological potential, in both in vitro and in vivo animal models, phlorotannins should not be discounted.

## Figures and Tables

**Table 1:** Antidiabetic activity of isolated phlorotannins *.

Compound	Source	Activity	Reference
6,6′-Bieckol	*Ecklonia cava* *Ecklonia bicyclis*	α-Glucosidase inhibition AR inhibition Protection against GIOS ↓Glucose-induced β-cells damage	[29,30,31]
8,8′-Bieckol	*Ecklonia bicyclis*	AR inhibition	[30]
DDBT	*Sargassum patens*	α-Glucosidase inhibition α-Amylase inhibition	[32]
Dieckol	*Ecklonia cava* *Ecklonia stolonifera* *Eisenia bicyclis*	α-Glucosidase inhibition α-Amylase inhibition ↓ Postprandial hyperglycaemia ↓ Serum insulin ↓ G6P AR inhibition PTP1B inhibition ↑ GLUT4 expression Inhibition of AGEs formation Protection against GIOS ↓Glucose-induced β-cells damage ↑ Glucose uptake Akt up-regulation ↓ C-peptide ↑ Adiponectin	[29,30,33,34,35,36,37,38,39]
Dioxinodehydroeckol	*Ecklonia stolonifera* *Eisenia bicyclis*	α-Glucosidase inhibition α-Amylase inhibition AR inhibition	[34,37,40]
Diphlorethohydroxycarmalol	*Ishige okamurae*	α-Glucosidase inhibition α-Amylase inhibition ↓ Postprandial hyperglycaemia Protection against GIOS ↓Glucose-induced β-cells damage	[41,42]
Eckol	*Eisenia bicyclis* *Ecklonia stolonifera* *Ecklonia cava*	α-Amylase inhibition AR inhibition PTP1B inhibition Inhibition of AGEs formation	[33,34,37]
Fucodiphloroethol-G	*Ecklonia cava*	α-Glucosidase inhibition	[29]
Fucofuroeckol-A	*Eisenia bicyclis*	α-Glucosidase inhibition α-Amylase inhibition	[40]
Hydroxybenzodioxin	*Eisenia bicyclis*	α-Amylase inhibition Inhibition of AGEs formation	[33]
Octaphlorethol-A	*Ishige foliacea*	α-Glucosidase inhibition ↓ Serum insulin ↓ G6P expression ↓PEPCK expression ↑Glut4 translocation/expression ↓Glucose-induced β-cells damage ↑ AMPK activation ↑ Glucose uptake	[43,44,45,46]
2-Phloroeckol	*Ecklonia stolonifera*	AR inhibition	[37]
7-Phloroeckol	*Ecklonia cava* *Ecklonia stolonifera* *Eisenia bicyclis*	α-Glucosidase inhibition α-Amylase inhibition AR inhibition PTP1B inhibition Inhibition of AGEs formation	[29,33,34,37]
Phlorofucofuroeckol-A	*Ecklonia cava* *Ecklonia stolonifera* *Eisenia bicyclis*	α-Glucosidase inhibition α-Amylase inhibition ↓ Postprandial hyperglycaemia AR inhibition PTP1B inhibition Inhibition of AGEs formation	[29,30,34,37,47]
Phloroglucinol	*Ecklonia stolonifera* *Eisenia bicyclis*	α-Glucosidase inhibition α-Amylase inhibition AR inhibition Inhibition of AGEs formation	[34,37,48,49]

* AGEs: advanced glycation end-products; Akt: protein kinase B; AMPK: AMP-activated protein kinase; AR: aldose reductase; DDBT: 2-(4-(3,5-dihydroxyphenoxy)-3,5-dihydroxyphenoxy) benzene-1,3,5-triol; G6P: glucose-6-phosphatase; GIOS: glucose-induced oxidative stress; GLUT4: glucose transporter 4; PEPCK: phosphoenolpyruvatecarboxykinase; PTP1B: protein tyrosine phosphatase 1B.

**Table 2:** Antidiabetic activity of phlorotannin extracts *.

Extract	Source	Activity	Reference
Acetone	*Fucus vesiculosus*	↓ AGEs formation	[49]
Dieckol-rich extract	*Ecklonia cava*	↓Postprandial hyperglycaemia ↓ Serum insulin ↑Glucokinase activity ↓ G6P activity	[51,52]
Ethyl acetate	*Sargassum aquifolium*	α-Glucosidase inhibition α-Amylase inhibition ↓Postprandial hyperglycaemia	[53]
Ethyl acetate	*Ecklonia stolonifera*	AR inhibition	[37]
Fucophloroethol-rich extract	*Fucus distichus*	α-Glucosidase inhibition α-Amylase inhibition	[54]
Methanol	*Sargassum ringgoldianum*	α-Glucosidase inhibition α-Amylase inhibition ↓Postprandial hyperglycaemia	[55]
Methanol	*Sargassum polycystum*	↓ AGEs formation	[56]
Methanol	*Spatoglossum asperum*	α-Glucosidase inhibition α-Amylase inhibition	[57]
Methanol	*Padina pavonica*	↓ AGEs formation	[56]
Methanol	*Turbinaria ornata*	↓ AGEs formation	[56]
Methanol (80%)	*Ishige okamurae*	↓ Blood glucose levels ↑ Insulin sensitivity	[58]
Water:acetonitrile (50:50 *v*/*v*)	*Ascophyllum nodosum*	α-Glucosidase inhibition α-Amylase inhibition	[59]
Water/Ethanol	*Ascophyllum nodosum* *Fucus spiralis*	α-Glucosidase inhibition α-Amylase inhibition	[60]
Water:methanol (25:75 *v*/*v*)	*Padina pavonica*	α-Glucosidase inhibition α-Amylase inhibition	[48]
Water	*Ascophyllum nodosum* *Fucus vesiculosus*	α-Glucosidase inhibition α-Amylase inhibition ↓ Postprandial hyperglycaemia ↓ Serum insulin ↓ Insulin secretion ↑ Insulin sensitivity	[61]

* AGEs: advanced glycation end-products; AR: aldose reductase; G6P: glucose-6-phosphatase.
